# Acute Application of Imidacloprid Alters the Sensitivity of Direction Selective Motion Detecting Neurons in an Insect Pollinator

**DOI:** 10.3389/fphys.2021.682489

**Published:** 2021-07-08

**Authors:** Elisa Rigosi, David C. O’Carroll

**Affiliations:** Department of Biology, Lund University, Lund, Sweden

**Keywords:** pesticides, motion detection, contrast sensitivity, insect vision, lobula plate tangential cells, nicotinic acetylcholine receptors, neonicotinoid

## Abstract

Cholinergic pesticides, such as the neonicotinoid imidacloprid, are the most important insecticides used for plant protection worldwide. In recent decades, concerns have been raised about side effects on non-target insect species, including altered foraging behavior and navigation. Although pollinators rely on visual cues to forage and navigate their environment, the effects of neonicotinoids on visual processing have been largely overlooked. To test the effect of acute treatment with imidacloprid at known concentrations in the brain, we developed a modified electrophysiological setup that allows recordings of visually evoked responses while perfusing the brain *in vivo*. We obtained long-lasting recordings from direction selective wide-field, motion sensitive neurons of the hoverfly pollinator, *Eristalis tenax*. Neurons were treated with imidacloprid (3.9 μM, 0.39 μM or a sham control treatment using the solvent (dimethylsulfoxide) only. Exposure to a high, yet sub-lethal concentration of imidacloprid significantly alters their physiological response to motion stimuli. We observed a general effect of imidacloprid (3.9 μM) increasing spontaneous activity, reducing contrast sensitivity and giving weaker directional tuning to wide-field moving stimuli, with likely implications for errors in flight control, hovering and routing. Our electrophysiological approach reveals the robustness of the fly visual pathway against cholinergic perturbance (i.e., at 0.39 μM) but also potential threatening effects of cholinergic pesticides (i.e., evident at 3.9 μM) for the visual motion detecting system of an important pollinator.

## Introduction

Major ongoing debate concerns the off-target effects on animal populations of widely used agrichemicals including neonicotinoid pesticides, such as imidacloprid (recently reviewed in bees in Alkassab and Kirchner, 2017; Wood and Goulson, 2017; Sgolastra et al., 2020). While targeted at pest species, beneficial arthropods such as pollinating insects are also exposed to these potential threats, both directly (via nectar and pollen of treated plants), and indirectly through exposure at sites of accumulation (e.g., soil, water, nests) (Alkassab and Kirchner, 2017). Neonicotinoids, including imidacloprid (IMI), act as partial agonists of nicotinic acetylcholine receptors (nAChRs) at synapses in the insect nervous system (Liu and Casida, 1993; Matsuda et al., 2001). Yet despite being commercially available for more than 20 years, surprisingly little is known about how sub-lethal doses of these chemicals affect the insect nervous system function particularly in intact individuals (Cabirol and Haase, 2019).

Prior work on the neurophysiological effects of these chemicals have primarily utilized cultured cells and isolated brain preparations (see for example: Buckingham et al., 1997; Déglise et al., 2002; Jepson et al., 2006; Barbara et al., 2008; Palmer et al., 2013; Wilson et al., 2013; Benzidane et al., 2017). While these *in vitro* approaches certainly help in identify effective sites of action, they provide little information on how neuronal function is affected in the whole living organism in the presence of relevant external sensory stimuli. Until recently, there had been only few attempts to redress this deficiency *in vivo*, for example, a calcium imaging study recorded odor-evoked responses from the antennal lobe of the intact honey bee and revealed impaired odor processing when the brain was exposed to an acute dose of IMI (Andrione et al., 2016).

The dearth of studies on the effects of IMI on the visual system is surprising given that many parts of the insect visual system are known potential targets for cholinergic agonists. Both cholinergic neurons and nAChRs are widely expressed in the insect optic lobes – both peripherally and centrally (Kreissl and Bicker, 1989; Brotz et al., 2001; Sinakevitch and Strausfeld, 2004; Raghu et al., 2011). Direct evidence for potential effects on visual processing includes acute exposure to sub-lethal doses of IMI, which caused increased cell death in the optic lobes of honey bees (de Almeida Rossi et al., 2013). More recently, visually-evoked responses recorded in visual pre-motor neurons from the locust ventral nerve cord showed impaired burst activity and activity propagation in animals previously injected or orally treated with a sub-lethal dose of IMI or two main secondary metabolites (Parkinson et al., 2017, 2020; Parkinson and Gray, 2019). Very recently Martelli and colleagues provided evidence of decreased synaptic transmission as well as phototransduction in photoreceptors of *Drosophila* chronically treated with imidacloprid (Martelli et al., 2020). These studies are of particular interest as they represent the first evidence of impairment *in vivo* in the visual nervous system of insects due to IMI exposure, although which neurons along the visual system are directly affected by imidacloprid, remains unknown.

At a behavioral level, honey bees fed orally with sub-lethal doses of IMI show alterations of complex visually-guided behaviors such as spatial learning, navigation and homing flights (reviewed in Alkassab and Kirchner, 2017). While these studies do not directly address individual visual neural circuits, throughout these behaviors, flying insects need to stabilize their head and body position during flight maneuvers and course control. In dipteran flies, this visually driven flight control is mediated by a well-studied class of neurons in the 3rd optic ganglia, the motion-sensitive lobula plate tangential cells (LPTCs, Busch et al., 2018). Both *in vitro* pharmacological studies and immunohistochemistry have showed that these classes of wide-field direction sensitive neurons in the fly are activated by ACh and express nAChRs in proximity of their dendrites (Schmid, 1992; Brotz and Borst, 1996; Brotz et al., 2001).

Given their well-described role during flying maneuvers and their association with the ACh system, we hypothesized LPTCs as a likely site of action of cholinergic pesticides. To test this, we developed a new preparation that allows us to record extracellularly or even intracellularly from LPTCs in the hoverfly, *Eristalis tenax* while perfusing the brain hemolymph with sub-lethal doses of IMI and during subsequent washout. Our preparation leaves the animal largely intact, allowing us to stimulate the eye with wide-field directional stimuli comprised of moving visual gratings and directly quantify the effects of IMI or its vehicle on electrophysiological responses of LPTCs. We found that when exposed to high doses of imidacloprid (3.9 μM) these cells showed increased spontaneous acitivity and a decrease in both contrast sensitivity and direction selectivity compared either to their normal state or to animals exposed to a control stimulus. Our new preparation thus on the one hand identifies a robustness of these flies to modest doses (observed at 0.39 μM) and potential effects at higher doses (observed at 3.9 μM) on the processing of directional motion in a species which is an important pollinator in its own right. Our approach also provides a new method for ongoing analysis of other visual pathways or in other species that should examine possible links between sub-lethal doses of neonicotinoids or other agrichemicals and visual function.

## Materials and Methods

### Experimental Design

**FIGURE 1:**
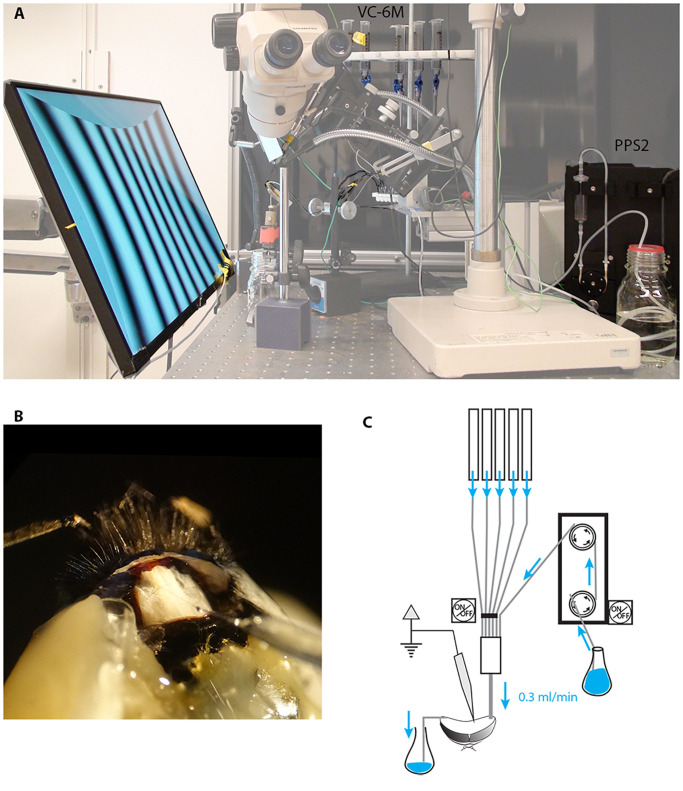
Electrophysiological set up for long-lasting recordings in perfused insects. We modified our existing set up to perform electrophysiological recordings while constantly perfusing the insect head capsule. **(A)** A low-noise peristaltic perfusion pump was fed into a micro ML-6 manifold apparatus that received also 5 PE-50 tubing from a 6-valve computer-controlled gravity system, each of these were connected to a 20 ml syringe filled with treatment solutions. **(B)** Close up of the insect head showing the inlet tubing, the outlet and the recording electrode (the example shown is in the bumblebee *Bombus terrestris* rather than *Eristalis*). **(C)** Schematic drawing of the perfusion system.

We built an *in vivo* perfusion system (Figure 1) to be able to constantly perfuse and expose the insect brain to two sub-lethal doses of imidacloprid (0.39 and 3.9 μM) and at the same time perform long-lasting electrophysiological recordings from lobula plate tangential cells (LPTCs) of an intact insect pollinator.

### Animal Preparation

Hoverflies [*Eristalis tenax* (Linnaeus, 1758)] were either collected outdoors or bought as pupae from Polyfly SL (Almeria, Spain). After animal collection on flowers, flies were taken into the lab and if not immediately used, individual flies were kept at 4°C in plastic bags with wet tissue and granulated brown sugar for up to 10 days. Flies maintained in the fridge for longer than 2 days were brought to room temperature for ∼1 h every 48 h to allow them to feed and clean themselves and the wet tissue with sugar replaced. Flies that emerged in the lab from pupae were used when they were 15–30 days old and fed *ad libitum* with brown sugar on a wet tissue at room temperature. Immediately before the experiment the insect was further fed with granulated brown sugar on a wet tissue.

Flies were inserted into a pipette tip, cut at its narrow end to approximate the diameter of the head, and the thorax and mouthparts fixed with low-temperature melting compound of 1:1 wax:rosin, tilting the insect head about 45° forward to optimize neural recording from the lobula plate tangential cells. First, the reference electrode was inserted at the base of the right eye. Then ∼150 mm length of thin polyethylene tubing PE-50 (outside diameter: 0.97 mm, inside diameter: 0.58 mm; Warner instruments, Harvard apparatus) was fixed with wax along the thorax so that the tip was able to perfuse the back of the eye. The wax:rosin compound was used to fill the gaps between the edge of the eye and the thorax in both sides in order to prevent the perfusion liquid to run off the head capsule. Subsequently, the cuticle from the back of the head was gently removed, fat bodies and tracheae removed with tissue paper and forceps. As soon as the brain was exposed the animal was put into the electrophysiological set up, the perfusion tubing connected (see below) and the recording inserted within 1 min, to avoid desiccation.

### Perfusion System

A low-noise peristaltic perfusion pump (PPS2, Multi Channel Systems MCS GmbH, flow rate: 0.3 ml/min) was fed into a micro ML-6 manifold (Warner Instruments, Harvard apparatus) that received also 5 PE-50 tubing from a 6-valve computer-controlled gravity system (VC-6M Valve Control System, Warner Instruments, Harvard Apparatus). Each input was connected to a 20 ml syringe filled with treatment solutions (Figure 1). Each of the channels of the gravity system was height adjusted to match the flow rate of the peristaltic pump (0.3 ml/min). The outlet of the micro manifold was then connected to the animal’s head through the PE-50 tubing as described above.

In our initial experiments with the suction-system provided by the PPS2 pump system to maintain a constant level of fluid in the head capsule, we found both vibration and electrical noise to be problematic as the suction system cyclically made and broke contact with the meniscus in the perfused area. Subsequently we solved this problem to maintain a constant fluid level in the head capsule through a capillary-based system, whereby we placed the tip of a thin cotton thread on the right side of the head with the other extremity in a glass container to collect the liquid (Figure 1). The constant capillary action in this thread gently draws fluid away from the perfused area at a rate high enough to allow the entire volume of the interior head capsule to be changed approximately every 10 s (0.3 ml/min). After experimenting with several different threads for this purpose, we found that a pre-washed and wetted tea-bag string (Twinings, United Kingdom) provided an ideal compromise between size and capillary action.

### Drug Delivery

Due to its low solubility in water, a stock solution of imidacloprid (Sigma-Aldrich Sweden AB) was obtained by dissolving 1 mg of imidacloprid (IMI) in 1 ml dimethylsulfoxide (DMSO, Sigma-Aldrich Sweden AB) and maintained at −20°C. On the day of each experiment the stock solution (IMI 3.9 mM) or DMSO alone were then diluted (1:1000) in insect Ringer solution comprising (in mM) the following: NaCl (140), KCl (10), NaH_2_PO_4_ (4), Na_2_HPO_4_ (6), CaCl_2_(H_2_O)_2_ (3), sucrose (90), and adjusted to pH 6.8.

A concentration of 3.9 μM (IMI_H_) was initially chosen on the basis of the EC50 value obtained in previous experiments in isolated insect brains and cultured neurons from honey bees and cockroaches (Buckingham et al., 1997; Déglise et al., 2002; Barbara et al., 2008; Palmer et al., 2013). A second concentration of imidacloprid (IMI_L_, 0.39μM) was subsequently tested (diluting the stock solution 1:10,000) to test the effects at a lower concentration. These concentrations appear to be sub-lethal, since we could still observe neural activity at the end of the experiments (even in experiments where we lost contact with the initial units recorded).

### Electrophysiological Recordings and Visual Stimuli

Extracellular recordings were performed at room temperature (23–26°C) using aluminosilicate glass capillaries (SM100F-10, Harvard Apparatus, Holliston, MA, United States) pulled with a P-2000 laser puller (Sutter Instruments, Novato, CA, United States) and filled with 1M KCl solution. The tip of the capillary was gently broken on fine 1000 grit carborundum paper to obtain a resistance of 1–10 MΩ and subsequently inserted in the lobula complex via a micro-manipulator (Marzhauser-Wetzlar PM-10; step size, 5 μm). The extracellular recordings were digitized at a 10 kHz sample rate, after hardware filtering with low-pass (3 kHz) and high-pass (3 Hz) filters built into the differential preamplifier (BC-03x, NPI, Germany).

**FIGURE 2:**
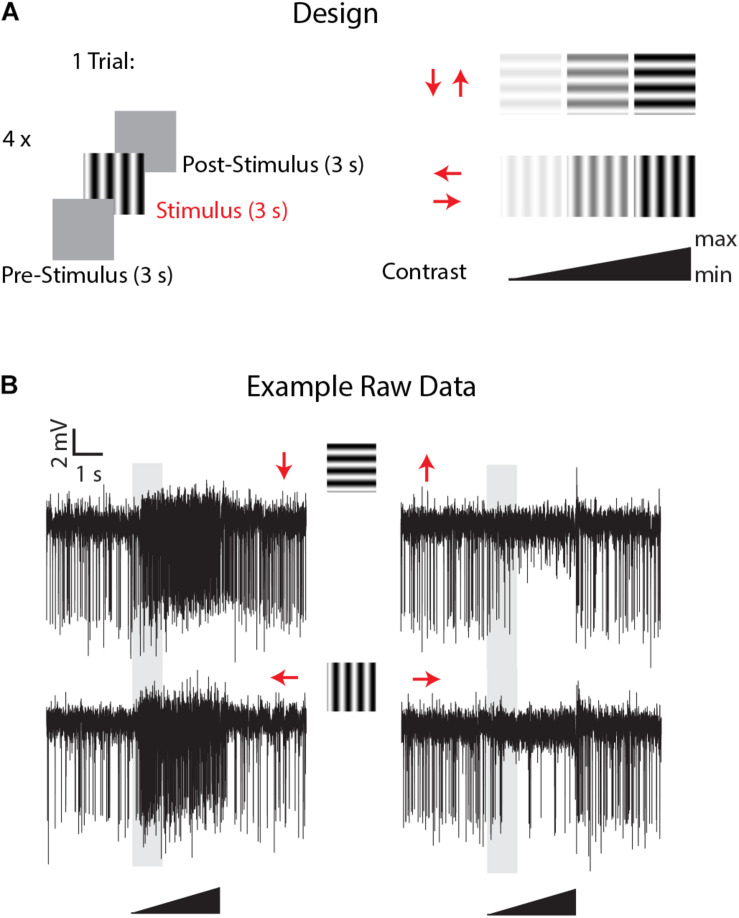
Experimental design and example of LPTCs direction selective responses in *Eristalis tenax* with ongoing perfusion. **(A)** the experimental design consisted of the following visual stimuli: a mean luminance (gray) screen left for 3 s (pre-stimulus period) was followed by a full-screen visual grating stimulus (3 s) in one of the 4 cardinal directions (Temporal frequency: 8 Hz; Spatial frequency: 0.1 cycles/deg) and then the mean luminance screen again for 3 s (post-stimulus response). Four different stimuli with different directions were tested in one trial, and the contrast of each visual stimulus consisted of a linearly increasing ramp of contrast from 0 to a maximum of either 0.5 or 1.0 (see methods for max values). **(B)** Time- course of visually-evoked responses of a trial in a recorded cell when the head of the animal was perfused with Ringer. Red arrows represent the direction of motion of the stimulus used; the 3 s visual stimulus is represented by the black triangle and the shaded area represents the analyzed time-window.

The animal sat in front of a high luminance, high speed IPS LCD monitor (Asus ROG Swift PG279Q, 2560 × 1440 pixels; ∼380 cd/m^2^; 144 Hz) and wide-field motion-sensitive neurons were identified on the basis of their characteristic opponent responses to wide-field motion, with maximal excitation for one direction and inhibition by the opposite direction (Figure 2) as well as their weak responses to smaller features. Because of the type of recordings (extracellular) we could only select the direction-sensitive cells on the basis of their spiking activity (see for example in Hausen, 1982) with no regards to graded changes in membrane potentials. Nevertheless the recording electrode tip location, frontal receptive fields, relatively high spontaneous rates, responses to motion stimuli and preferred direction (leftwards or downwards) were all consistent with recordings from vertical and horizontal system neurons of the lobula plate (Hausen and Egelhaaf, 1989). Visual stimuli were presented using custom-written software implemented in Matlab (The MathWorks, Natick, MA, United States) and Psychtoolbox (psychtoolbox.org), with calibration for gamma correction. Once the position of the electrode on the brain was identified as suitable for the targeted neurons the perfusion system (with Ringer solution) was turned on so that the head capsule was filled. At the same time the cotton string was carefully added laterally on the right part of the head to suck the solution away and maintain a constant fluid level within the head volume. Once the liquid in the head capsule was rising and in contact with the capillary containing the electrode, the electrical contact was sometimes lost and a good new single unit recording had to be re-established by moving the electrode a few microns.

Experiments commenced once we had established a clear and stable single or multiunit response from a wide-field motion-sensitive cell with the perfusion system on. Useful recordings then lasted up to 3 h using the same recorded units. Stimuli comprised a mean luminance (blank) screen for 3 s (pre-stimulus period), stimulated with a full-screen visual grating stimulus (Temporal frequency: 8 Hz; Spatial frequency: 0.1 cycles/deg) for 3 s and then a return to the mean luminance screen for 3 s (post-stimulus response, Figure 2). To give an indication of contrast sensitivity, the contrast of each grating stimulus linearly increased from a Michelson contrast of 0 to 1, except in 3 cells in which the maximum contrast was kept lower (0.5) to avoid response saturation due to the high contrast sensitivity of these cells. Four directions were tested in 4 different sequential trials (inter-trials interval: 0 s). This direction experiment was replicated about every 7 minutes. 30–40 min after the experiment started the perfusion system was switched to the treatment (via the multi-valve gravity system; main pump (PPS2) with Ringer set to off, see Figure 1) and the direction selectivity tested 5 min after the perfusion with treatment started. The treatment lasted for a maximum of 1 h, after which the main peristaltic pump with Ringer alone was switched back again (multi-valve gravity system off) for 45 min to washout the treatment in the head capsule and the direction stimuli again tested every 7 min through this period.

### Data Analysis and Statistics

**FIGURE 3:**
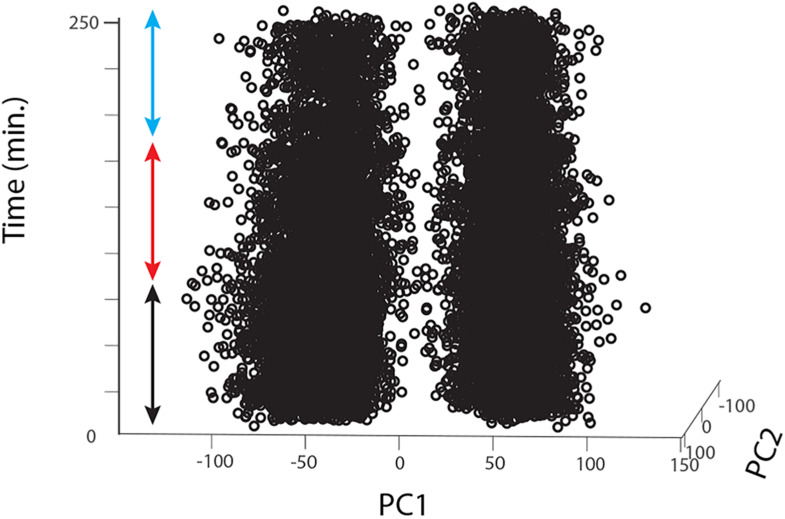
Long-lasting recordings show long-lasting stability in the 2D feature space of the detected spikes. The figure shows an example for one fly treated with DMSO. Spikes detected using Offline Sorter software are represented as single dots (in this case for 2 different units) plotted against the first 2 principal components of the feature space that explains their main waveform differences and plotted over time. The colored arrows indicate the pre-treatment (black), during treatment (red) and washout (cyan) periods.

For each animal, raw data were imported into Matlab, digital filtered with a band-pass filter (2nd order Butterworth, 125 Hz, 1.25 kHz low and high corner frequency, respectively), we then concatenated all Matlab raw data files into a single vector that we imported into Offline Sorter v4 (Plexon Inc, Texas, United States). The concatenation process allowed us to include a larger number of detected events (i.e., multiunit spikes) and thus more statistical power to perform a better unit sorting than on single trials. In Offline Sorter, we applied a double spike detection threshold of either ± 4 or 4.5 SD depending on the SNR of the individual preparation. Units were then identified as separated clusters in the 3D PCA space using a manual quality-controlled approach after initial automatic sorting (Valley Seeking algorithm, Parzen multiplier 0.8–1) and after realigning waveforms to their global maximum. 2 to 3 motion-sensitive units were thus identified from each recording (single animals); for each unit we then applied an inclusion criterion for further analysis only for ‘direction opponent’ units that also showed inhibitory responses to anti-preferred direction motion, i.e., responses lower than spontaneous activity evaluated in the pre-stimulus condition (before drug/sham application). From 20 flies we thus obtained 12, 8, and 12 such units for the DMSO, 0.39 μM IMI and 3.9 μM IMI treated flies, respectively. Finally, we controlled for the stability of the recordings in the DMSO and in all pre-treatment trials (before drug/sham application) by plotting the first 2 principal components for all spikes against time. We only included units that showed no clear changes or drifts in the variability of the detected waveforms (example for a DMSO fly in Figure 3).

**FIGURE 4:**
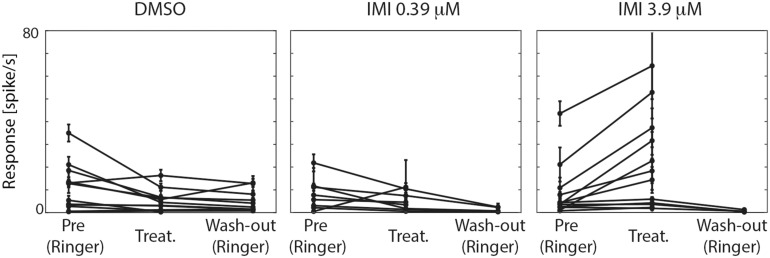
High (3.9 μM) but not low (0.39 μM) doses of imidacloprid (IMI) increases the spontaneous responses of lobula plate tangential cells (LPTCs) of the pollinator *E. tenax*. Average spontaneous responses (spike/s) ± 95% CI for all the recorded units are shown for all the conditions within each treatment.

**FIGURE 5:**
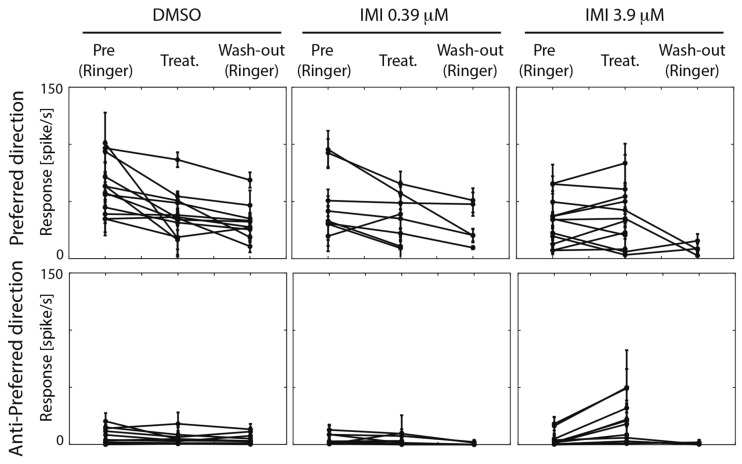
High (3.9 μM) but not low (0.39 μM) doses of imidacloprid alter direction selectivity of LPTCs in the fly pollinator *E.tenax*. Average response ± 95% CI for all treatment and conditions for preferred and anti-preferred stimuli. Each point represents one unit. Replicates per unit: 2–10.

For each experimental condition, we calculated the averaged responses (spike/s; average of 2–10 technical replicates for each condition) along all periods before visual stimulus presentation (i.e., spontaneous responses, Figure 4) and during visual stimulation (preferred and anti-preferred stimuli, Figure 5). To avoid confounding the response to contrast with motion adaptation to the visual stimulus over time (see for example Nordström et al., 2011), we calculated the responses to visual stimuli in the very low contrast range (0.3–17% contrast, see gray area in Figure 2 and averaged responses in Figure 5). Direction selectivity was calculated as an index as follows: DI = (P-AP)/(P+AP), with P and AP referring to the calculated responses for preferred and anti-preferred stimuli, as described above. Contrast sensitivity was calculated by averaging spiking frequency along the linear contrast ramp response in bins of 150 or 320 ms (for stimuli with a maximum contrast of 1 and 0.5, respectively) corresponding to bands of contrast 0.06 wide, in the range between 0 and 0.5. For each response in the preferred direction a Weibull function was fitted to the data using a simplex search algorithm to obtain the best-fit values of output range and slope. Since the Weibull function scales on the basis of the absolute values of the responses, we normalized responses for each trial based on the averaged low contrast response (contrast: 0.3–28%) for each unit during the pre-treatment condition.

We analyzed contrast gain by measuring C50, the contrast needed to evoke a 50% maximal response based on fitting a Weibull function to the data (Nordström et al., 2011). Contrast gain reduction would be seen as an increase in the response required to reach this threshold.

Expected changes in contrast sensitivity are characterized by two different and independent changes in the curve: a horizontal shift (described by the “alpha” parameter in the Weibull function) in the initial contrast threshold that indicates the contrast gain and a change in the response gain of the curve (described by the “gain” parameter of the Weibull fit) that determines the final output range for high contrast patterns. A full clarification of these different components of motion adaptation is given in Nordström et al. (2011).

To interpret our data we used an approach based on effect size and estimation statistics.^1^ For spontaneous responses, directionality indices, preferred responses and contrast sensitivity curves, pair mean differences (before and after treatments) were estimated calculating the unbiased Cohen’s *d* and bias-corrected 95% CI using the ESCI module (Exploratory Software for Confidence Intervals)^1^ using the free, open-source software Jamovi.^2^ For each comparison we also provided null-hypothesis based *p* values using Wilcoxon signed Rank Test corrected for multiple comparisons (Bonferroni correction), with our new *p* threshold being *p* = 0.0125. Spontaneous responses were tested across conditions using Kruskal-Wallis Test to compare among treatments (IMI high-IMI low-DMSO). These tests were performed in Matlab.

## Results

### Long-Lasting Extracellular Recordings From the Insect Lobula in Constantly Perfused Animals

Our apparatus used a peristaltic pump combined with a computer-controlled multi valve gravity fed system to supply artificial haemolymph to the head capsule of the insect (Figure 1). This system allows for either intracellular or extracellular recordings from the insect brain while continuously perfusing with a main flow (usually Ringer solution) and up to 5 different treatments to switch into this flow at a relatively high flow rate (0.3 ml/min). The success and duration of the recordings in these conditions depend upon the size and type of neurons targeted for recording, the proximity of the electrode tip to the inlet of the perfusion system and on the type of recordings (intra/extracellular). We used this setup to record from motion-sensitive lobula plate neurons of the hoverfly, *Eristalis tenax*, and investigate how their physiological response to 4 different directional stimuli (Figure 2) was affected by the exposure to sub-lethal doses of imidacloprid.

Our application of a low-vibration pump to supply the main saline flow and our novel implementation of a vibration-free capillary suction system permitted brief intracellular recordings from lobula plate neurons (data not shown). However, we found that extracellular recordings from the same class of neurons allowed for more stable and much longer-lasting recording sessions, up to 3 h in healthy cells. This allowed us to obtain visual-evoked responses during a resting state (pre-treatment), during the treatment and during subsequent washout of the treated agent, all within the same unit. However, even with our low-vibration delivery and suction system, this type of recording remains highly challenging and the success rate (long-lasting, healthy recording with good signal-to-noise ratio, see for example Figures 2, 3) was only in 1 out of 3 preparations. While the healthy recordings with the vehicle were able to last through the subsequent washout (∼ 3 h) in all cases except one, we lost reliable electrophysiological contact with the same units after treatment in 8 out of 13 animals treated with IMI, most likely due to additional disturbance following the typical motor responses invoked by this agent.

### Imidacloprid Can Increase Spontaneous Responses of LPTCs

As in prior studies we used DMSO as a solvent to prepare stock IMI solutions because of IMI’s low aqueous solubility. DMSO has, however, been shown to cause cellular toxicity through plasma membrane pore formation, which might alter neuronal signaling (Galvao et al., 2014). Surprisingly, as far as we are aware, effects of DMSO have not been extensively quantified in behavioral or neurophysiological experiments when testing pesticides. However, decreased neural activity due to DMSO has been reported: high concentrations of DMSO cause silencing of responses in sensory neurons in *Locusta migratoria*, for example (Theophilidis and Kravari, 1994), as well as inducing changes in the permeability of neuron membranes, for example in the visual system (Weckström and Laughlin, 2010).

We therefore first tested whether the spontaneous firing rate of the neurons was affected by treatment exposure. In the pre-treatment condition, the three groups showed no significant difference in spontaneous spike rates (Kruskal Wallis test, *p* = 0.7577, Figure 4). Following exposure to either IMI 3.9 μM or to its vehicle, DMSO, significant changes in spontaneous activity were observed, but in opposite directions: IMI 3.9 μM increased the spontaneous firing rate with a medium-large effect size (corrected Cohen’s *d* 0.70, 95% CI [0.40, 1.18], *p* = 0.0015 Wilcoxon signed Rank Test), while the vehicle, DMSO, decreased it (corrected Cohen’s *d*: −0.70, 95% CI: [−1.31, −0.23], *p* = 0.0269 Wilcoxon signed Rank Test). Lower dose (0.39 μM) IMI had no effect on spontaneous activity (corrected Cohen’s *d* −0.48, 95% CI [−1.45, 0.33], *p* = 0.1484 Wilcoxon signed Rank Test, Figure 4).

We then asked whether the absolute response of the cell to the preferred stimulus changes among conditions. Because responses among units were variable prior to treatment, we only performed analyses before and after exposure within each of the treatments. When comparing pre (Ringer) and responses during treatment in the same unit within each condition (estimated pair mean difference), DMSO caused a large decrease in the motion responses (corrected Cohen’s *d* −1.07, 95%CI [−1.92, −0.40] *p* = 0.0015, Wilcoxon Signed Rank Test). Low dose (0.39 μM) IMI also caused a small decrease in responses to preferred direction motion, (corrected Cohen’s *d* −0.5, 95% CI [−1.11 −0.06], *p* = 0.0547 Wilcoxon Signed Rank Test) although confidence limits include a possibility that this is a negligible effect. Finally, high dose (3.9 μM) IMI had no measurable effect on responses (corrected Cohen’s *d* 0.1, 95% CI [−0.25, 0.47], *p* = 0.5186 Wilcoxon Signed Rank Test). Taken together, these results suggest that the vehicle (DMSO) indeed dampens the motion response, although there is a partial reversal of this effect with high dose IMI, possibly due to the increase in spontaneous (non-motion dependent) activity.

### High (3.9 μM) but Not Low (0.39 μM) Doses of Imidacloprid Altered Directional Tuning and Contrast Sensitivity in LPTCs

**FIGURE 6:**
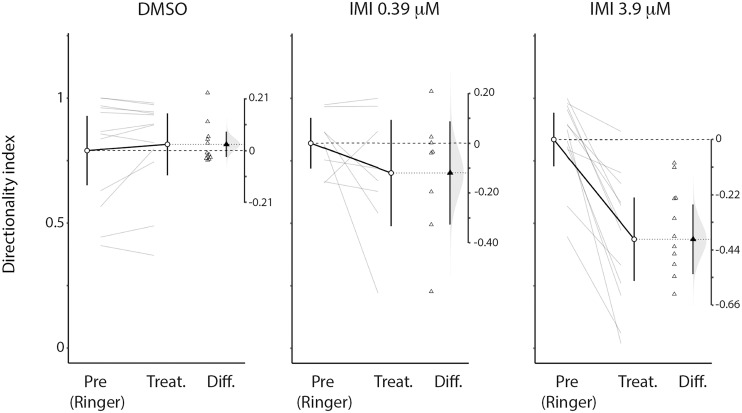
High (3.9 μM) but not low (0.39 μM) doses of imidacloprid alter direction selectivity of LPTCs in the fly pollinator *E.tenax.* Directionality index (see method section for calculation) plot for Pre- and all respective treatment conditions (DMSO, IMI_L_, IMI_H_). The differences of the indices are also reported together with the distribution of the bootstrap simulation to assess the confidence of our results (plots originated and exported from ESCI module in Jamovi software, www.jamovi.org).

An inclusion criterion for our classification of recorded neurons as LPTCs was that they were motion opponent i.e., their spontaneous activity was inhibited by stimuli in the anti-preferred direction (Figure 2). Interestingly, we observed a large increase in the response to anti-preferred direction stimuli only for the units exposed to 3.9 μM IMI (corrected Cohen’s *d* 0.98, 95% CI [0.65, 1.50], *p* = 0.0005 Wilcoxon signed Rank Test). This suggests that the general direction sensitivity of the neuron might have been affected. To investigate this further, we calculated a directionality index in each unit for every condition (Figure 6), whereby a value of 1.0 indicates complete inhibition by anti-preferred directions, and a value of 0 would indicate no difference between the preferred and anti-preferred responses. We observed a dramatic decrease in direction selectivity of cells treated with 3.9 μM IMI (corrected Cohen’s *d* −1.74, 95% CI [−2.17, −1.13], *p* = 0.0005, Wilcoxon signed Rank Test) but not in those treated with 0.39 μM IMI (corrected Cohen’s *d* −0.57, 95% CI [−1.59, 0.27], *p* = 0.250, Wilcoxon signed Rank Test) nor for DMSO alone (corrected Cohen’s *d* 0.12, 95% CI [−0.10, 0.35], *p* = 0.5693 Wilcoxon signed Rank Test).

**FIGURE 7:**
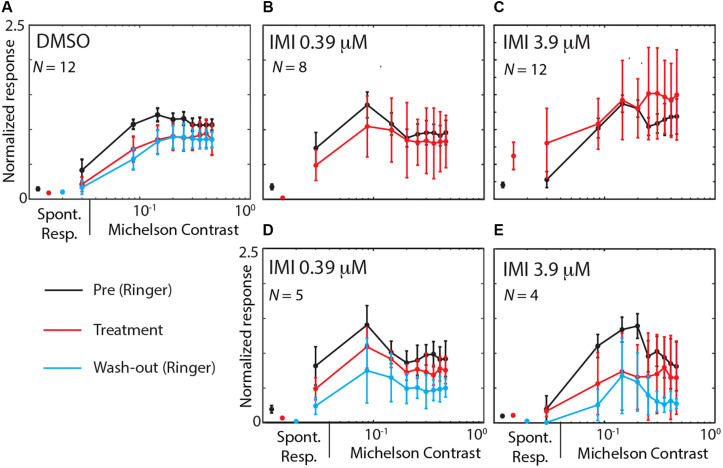
High (3.9 μM) but not low (0.39 μM) doses of imidacloprid disrupt contrast sensitivity of LPTCs in *E. tenax*. Normalized Average (see methods for details on normalization) ± 95% CI responses to different contrasts in units that are exposed to DMSO **(A)**, IMI_L_ **(B,D)** or to IMI_H_ **(C,E)**. Contrast sensitivity curve are showed during pre-treatment (perfused with Ringer, black lines) and during treatment exposure (red lines) for the preferred direction. Panels **(B,C)** show pre-treatment and treatment contrast responses in all units where IMI was used, while panels **(D,E)** only show data for experiments where we could also record responses during washout (cyan). On the left part of each figure spontaneous activity is shown as average ± 95% CI response of the same color as for each condition. Contrast values are mean values of contrast obtained binning approximately 0.06 contrast intervals and responses are averaged spike frequency in the corresponding time windows. Stated *N* values are the units used for each plot.

We finally asked whether IMI affects the contrast sensitivity of LPTCs. Each stimulus presented consisted of a linear ramp of increasing contrast (Figure 2), so responses that commence earlier within the presentation period indicate a higher sensitivity to the stimulus pattern (O’Carroll et al., 1997). This allowed us to analyze the contrast sensitivity of the response in the preferred direction by temporally binning the responses into small contrast ranges (Figure 7). We observed a medium decrease in the output range (response gain in the Weibull fit) for 3.9 μM IMI (corrected Cohens’*d* −0.57 95% CI [−1.16 −0.09], *p* = 0.034 Wilcoxon signed Rank Test) although neither DMSO (corrected Cohen’s *d* −0.38 95% CI [−1.28, 0.43], *p* = 0.6772 Wilcoxon signed Rank Test) nor 0.39 μM IMI (corrected Cohen’s *d* −0.28 95% CI [−0.80, 0.15], *p* = 0.3828 Wilcoxon signed Rank Test) caused a reliable reduction. We saw no effect of DMSO nor of 0.39 μM IMI of treatment on the contrast gain measured as C50 (DMSO, corrected Cohen’s *d* 0.4, 95% CI [−0.22 1.13], *p* = 0.0840 Wilcoxon signed Rank Test; 0.39 μM IMI: corrected Cohen’s *d* 0.43, 95% CI [−0.65 1.80], *p* = 0.3125 Wilcoxon signed Rank Test). However, we observed an increase in contrast gain (i.e., a lower threshold contrast) with a medium effect size when 3.9 μM IMI was applied (corrected Cohen’s *d* −0.55, 95% CI [−1.11 −0.1], *p* = 0.0186 Wilcoxon signed Rank Test). We note, however, that even at contrasts below threshold, responses are significantly elevated compared with control, so this could represent a non-motion dependent contribution to neuronal firing rather than an increase in contrast gain per se.

## Discussion

We used electrophysiological recordings combined with a peristaltic perfusion system to investigate the effects of sub-lethal doses of the widely used neonicotinoid, imidacloprid, on the wide-field motion sensitive cells of the pollinator fly *Eristalis tenax*. The fly lobula plate tangential cells (LPTCs) are a well-known model for the study of motion detection (recently reviewed in Silies et al., 2014; Yang and Clandinin, 2018; Borst et al., 2019). These neurons normally show increased activity when a wide-field moving stimulus is presented in a specific direction and are inhibited when the motion is presented in the opposite direction (see Figure 2). We found that when a high dose of imidacloprid (3.9 μM) was perfused into the head capsule of the animal, the recorded cells showed higher spontaneous responses (Figure 3) as well as responses to normally anti-preferred movements (Figure 5). This leads to both their directional selectivity and contrast sensitivity being altered, indicating a generally reduced ability to encode attributes of stimuli to which these cells are normally sensitive. Interestingly, DMSO caused a dramatic reduction on spontaneous responses (Figure 3). Acute exposure to lower doses of imidacloprid (0.39 μM) did not substantially alter the encoding of contrast or directional selectivity.

LPTCs get their inputs from direction-selective columnar neurons that process locally retinotopic motion signals from the periphery (Douglass and Strausfeld, 1996; Silies et al., 2014; Shinomiya et al., 2019). As has been theoretically described, direction opponent responses such as observed in Figure 2 arise from the computation derived from dendrites of LPTCs and their inputs where both inhibitory GABA and excitatory nAChR are present (Brotz et al., 2001; Sinakevitch and Strausfeld, 2004; Shinomiya et al., 2019; Davis et al., 2020). Indeed both GABA and nAChR are seen in close proximity of LPTCs (Brotz et al., 2001) and these are functionally activated by ACh and its agonists (Schmid, 1992; Brotz and Borst, 1996). Thus it seems plausible that in addition to any indirect impairment from other cholinergic neurons upstream from the LPTCs, the cholinergic pesticide imidacloprid might act directly on the LPTCs.

Imidacloprid at 0.39 μM doses did not significantly affect the stimulus-evoked responses in our case as seen previously in other visual neurons downstream to LPTCs in the locust (Parkinson et al., 2017; Parkinson and Gray, 2019). This might be explained by the fact that the EC50 curve that we used to choose our doses (Buckingham et al., 1997; Déglise et al., 2002; Barbara et al., 2008; Palmer et al., 2013) may not represent the EC50 curves for our neurons and more generally, our species, *Eristalis tenax*. Toxicological essays have been mainly performed in bees, with very few studies conducted on other pollinators. Among these, hoverflies have been completely overlooked, with only one study published recently (Basley et al., 2018) where higher doses of another neonicotinoid compound, thiamethoxan, reduced the survival rate of adults if the larvae were exposed to pesticides introduced into their aquatic environment. However, differences can also come from a different affinity of the nAChR in the LPTC pathway for imidacloprid compared to downstream neurons (Dupuis et al., 2011; Benzidane et al., 2017) or may be due to other parts of the visual system that might indirectly affect the response we record in LPTCs. Lamina, medulla and lobula neurons express nAChRs (Kreissl and Bicker, 1989; Brotz et al., 2001; Raghu et al., 2011) as well as a centrifugal modulation from lamina to photoreceptors that has been hypothesized to be glutamatergic or cholinergic (Zheng et al., 2006). This underscores the need for additional comparative analysis of the dose-response relationship in a range of pollinator species.

We also found that IMI 3.9 μM altered the contrast sensitivity of LPTCs, in particular reducing the range over which variations in the stimulus strength produce modulation of the response. These changes in output range after application of 3.9 μM IMI might be partially explained by a general increment in response activity and/or by an impaired contrast sensitivity in the upstream processes, or by other indirect mechanisms, for example through modulation of efferent octopaminergic neurons that have been shown to play a role in changing contrast output range of the animal (Jung et al., 2011). Either way, the reduced output range we observed indicate an effect on the ability of these neurons to encode relevant attributes of the input stimulus, whether it be the direction or the speed of a moving pattern.

An obvious question that arises from this kind of experiment is whether the doses we used are field-relevant. The highest concentration we used is comparable with imidacloprid EC50 values previously described in insect neurons. These values change on the basis of the preparation and type of neurons, but are in a range between 0.3 and 23 μM at least as described in the honey bee and in *Periplaneta* cholinergic neurons (Buckingham et al., 1997; Déglise et al., 2002; Barbara et al., 2008; Palmer et al., 2013). A limitation for us in selecting a field-relevant dose is the lack of studies assessing the toxicity of imidacloprid through this specific route of exposure, i.e., applied directly within the head capsule. However, Moffat et al. (2015) showed that imidacloprid reaches up to similar concentrations in the brain to the ingested source, within 3 days of chronic exposure (Moffat et al., 2015). A clear picture of what is a relevant field dose is nevertheless missing, with imidacloprid concentration in nectar and pollen found on site probably 2 orders of magnitude lower than the concentration we used (Blacquière et al., 2012). However, a similar concentration to the one we used was found, for example, in pollen stored in apiaries in 2010 (Mullin et al., 2010) and concentrations up to 2 orders of magnitudes higher than we used were measured in guttation drops of seed treated plants (Girolami et al., 2009). Nonetheless, these values might easily underestimate what the brain of the insect is exposed to considering multiple sources of exposure and accumulation. Furthermore this picture is complicated by the observation that the secondary metabolites during chronic exposure showed vastly higher toxicity than acute exposure to imidacloprid itself, no doubt contributing to the lethality of these chemicals in field exposure (Suchail et al., 2001). We have not yet tested such metabolites using our preparation, but they would be expected to act on the same receptors (Nauen et al., 2001; Palmer et al., 2013) and experiments applying some of these chemicals to the locust brain have recently shown them to act similarly to imidacloprid (Parkinson and Gray, 2019).

Finally, we should consider what effect the changes we observe might have on the animal’s behavior. While IMI likely has many other effects on other neuronal pathways than we observed in LPTCs, we can at least speculate as to possible behavioral impairments on the basis of what has been described for the role of these neurons in visually driven behaviors intrinsically linked to the detection of motion in specific local directions, e.g., information related to optic-flow, the perceived movement of the world driven by ego-motion. From LPTCs, direct connections project to motor neurons involved in neck and head movements, fundamental for flight-stabilization and course control (Gronenberg et al., 1995; Busch et al., 2018); as well as indirect projections involved in the looming-detection pathway important for avoidance behaviors (de Vries and Clandinin, 2012). The terminals of LPTCs also connect with descending visual interneurons that provide input to wing muscle motor neurons via a visually gated feedback circuit involving the mechanosensory halteres, thus combining input from both sensory modalities to control steering (Frye and Dickinson, 2001). Hence deficits in these pathways due to pesticide exposure might be expected to disrupt gaze and both head and body stabilization as normally driven by optical flow.

Moreover, motion-information sent through the posterior ventrolateral protocerebrum appears to be used by higher centers to accomplish behaviors such as landing, flight-speed regulation as well as “visual odometry” for measuring distances (Srinivasan et al., 1999). Studies of the LPTCs in *Eristalis* indeed reveal that non-linear adaptation in these neurons provides an excellent basis for robustly encoding the velocity of optic flow in natural scenes (Barnett et al., 2010), thus potentially contributing to the kind of visual odometry that plays a key role in navigation to and from the food source in a variety of insect species. Again, disruption to cholinergic signaling in this pathway might be expected to lead to errors in estimated distances and thus less efficient foraging. Moreover, motion information encoded in LPTCs has also been shown to be relevant for the detection of objects against a moving background, such as the one the insect experiences while flying through the environment (Fenk et al., 2014; Mertes et al., 2014). Using a behavioral assay in *Drosophila melanogaster*, for example, the retinotopic inputs to LPTCs (T4-T5 cells) have been shown to be necessary for object-fixation and discrimination against a moving background (Fenk et al., 2014). Even more convincingly, Mertes and colleagues (Mertes et al., 2014) recorded from motion-sensitive cells in the bumblebee while presenting visual stimuli reconstructed from their learning flights in an experimental arena. The visual evoked responses from these neurons indeed revealed their role in the detection of landmarks presented in the arena (Mertes et al., 2014). Interestingly, in the past, imidacloprid-induced impairments in honey bee navigation system have been reported: bees fed with sub-lethal doses of IMI showed longer or shorter foraging trips as well as incorrect homing flights (Schneider et al., 2012; Fischer et al., 2014; Gill and Raine, 2014; Colin et al., 2019; Muth and Leonard, 2019). Our findings thus suggest that the neurophysiological mechanisms involved in such impairments could easily begin with disruption to normal direction and contrast coding by neurons such as LPTCs.

To summarize, our data revealed a disrupted direction selectivity and contrast sensitivity in LPTCs after direct application of high doses of IMI to the brain, while the motion detection system seems to be robust against low dose application. At the same time we see a clear effect of DMSO in opposite directions to IMI, stressing the importance to take DMSO into account in further studies. We thus reveal a mechanism by which cholinergic pesticides might act in the brain of flying pollinators to affect motion-guided behaviors. Ultimately, our experimental setup will allow future investigation to identify where and how the visual system is affected by neonicotinoids and other pesticides under conditions in which the animals still respond to environmentally relevant sensory stimuli.

## Data Availability Statement

The raw data supporting the conclusions of this article will be made available by the authors, without undue reservation.

## Author Contributions

ER and DO’C: conceptualization, analysis, writing – review and editing, and funding acquisition. ER: experiments, visualization, and writing-original draft. Both authors contributed to the article and approved the submitted version.

## Conflict of Interest

The authors declare that the research was conducted in the absence of any commercial or financial relationships that could be construed as a potential conflict of interest.

## Footnotes

1 www.thenewstatistics.com

2 www.jamovi.org
